# Acute Gastroenteritis in the Etiology of Inflammatory Bowel Disease: Systematic Review and Meta-analysis

**DOI:** 10.1093/crocol/otab065

**Published:** 2021-10-01

**Authors:** Angelina Di Re, Yi Liang, Martijn Pieter Gosselink, Grahame Ctercteko

**Affiliations:** 1 Department of Colorectal Surgery, Westmead Hospital, Westmead, New South Wales, Australia; 2 School of Medicine, University of Sydney, Camperdown, New South Wales, Australia; 3 Department of General Surgery, Blacktown Hospital, Blacktown, New South Wales, Australia; 4 Department of Colorectal Surgery, Dr. Horacio E Oduber Hospital, Caya Punta Brabo, Aruba

**Keywords:** inflammatory bowel disease, gastroenteritis, etiology

## Abstract

**Background:**

Inflammatory bowel disease (IBD) consists of a spectrum of disorders including ulcerative colitis and Crohn’s disease, with a rising incidence worldwide. However, despite this prevalence the etiology of IBD remains uncertain. It has been suggested that an episode of gastroenteritis may precipitate IBD.

**Methods:**

Studies were identified using a literature search of Pubmed/Medline and Embase/Ovid. This review was performed according to the Preferred Reporting Items for Systematic Reviews and Meta-Analysis (PRISMA) guidelines. The primary outcome was incidence of new-onset IBD after gastroenteritis. Secondary outcomes included incidence of IBD after bacterial, viral, and parasitic gastrointestinal infections.

**Results:**

Eleven studies (*n* = 923 608 patients) were included. Four studies assessed patients with gastroenteritis, subsequently developing IBD as the primary outcome. Patients with gastroenteritis had a higher incidence of subsequent IBD but this did not reach statistical significance (odds ratio [OR] 3.81, 95% CI 0.52–27.85, *P* = .19). Seven studies examined the incidence of antecedent gastroenteritis (primary outcome) in patients with a confirmed diagnosis of IBD, compared to the controlled population. There was no difference between incidence of antecedent gastroenteritis across the 2 population groups (OR 1.07, 95% CI 0.55–2.08, *P* = .85). There was no association between IBD and bacterial, viral, or parasitic infections.

**Conclusions:**

In summary, our meta-analysis has shown that there is considerable heterogeneity in the literature regarding the role of gastroenteritis in the development of IBD. Further higher quality studies need to be performed to ascertain the true nature of this.

## Introduction

Inflammatory bowel disease (IBD) encompasses a spectrum of disorders including ulcerative colitis (UC), Crohn’s disease (CD), and unclassified.^1^ It is characterized by chronic relapsing gastrointestinal inflammation, and is associated with an increasing incidence worldwide.^2^ In the western world, the prevalence of IBD is 120–200/100 000 persons for UC, and 50–200/100 000 persons for CD.^3^ However, despite this prevalence, the etiology and pathogenesis of IBD remain uncertain.^4,5^

It is believed that IBD develops from an excessive immune response in genetically at-risk individuals.^6^ There is also a likely environmental trigger which affects the gut microbiota, although no definite trigger has been identified.^6,7^ Proposed risk factors for IBD include alterations in gut microbiota associated with antibiotic use, smoking, dietary fiber, saturated fats, impaired sleep, oral contraceptive pill use, and hormone replacement therapy.^8,9^ It has been suggested that certain gut bacteria may predispose patients to IBD.^10–13^ It has even been suggested that common gastroenteritis may be a causative factor in the initiation of IBD.^14,15^ Additionally, gastrointestinal infections are associated with exacerbations of colitis in patients with established IBD.^16,17^

We aim in this systematic review and meta-analysis to provide an updated assessment of the association between gastroenteritis and the development of IBD, as well as any associations with specific enteric pathogens.

## Materials and Methods

### Literature Review

This systematic review was registered with PROSPERO (International Prospective Register of Systematic Reviews). The PRISMA guidelines (Preferred Reporting Items for Systematic Reviews and Meta-Analyses) were used to assist in writing this review.^18^ The need for obtaining institutional review board approval or patient informed consent was waived for this study because it is a review of publicly available data.

### Search Strategy and Selection Criteria

The investigators designed and constructed the search strategy using relevant databases from commencement to April 17, 2019. Relevant studies were identified using a literature search of the following databases: Pubmed/Medline and Embase/Ovid. Published systematic reviews and meta-analyses (including their reference lists) were also searched from January 1, 1966 until April 17, 2019 to identify further studies. Two reviewers (A.D. and T.L.) individually assessed, included, and excluded studies to for this review. Search terms were filtered by “human” and “English,” and tailored to each database using the following Medical Subject Headings (MeSH; in bold) and key terms:

1. **Inflammatory bowel disease** OR **Crohn’s disease** OR **ulcerative colitis**
2. **Gastroenteritis** OR **gastrointestinal infections** OR **bacteria** OR **virus** OR **parasitic infection**
3. **Etiology**
4. #1 AND #2
5. #1 AND #2 AND #3

Prospective and retrospective studies were included in our review if they met the following criteria: (1) included IBD patients with a diagnosis of gastroenteritis, (2) studies published in English, (3) involved adults (≥18 years old), (4) involved humans, and (5) the full text of the article was available. If relevant information was not reported or if there were any queries, then the relevant study investigators were contacted to provide clarification.

Studies were excluded if they were (1) case studies or series (less than 10 patients), (2) animal studies, (3) presented data in a form unable to be analyzed, and (4) if the gastroenteritis episode did not precede IBD development.

### Outcomes of Interest

The primary outcome was incidence of IBD (UC, CD, or unclassified) after 1 or more episodes of gastroenteritis. Secondary outcomes included incidence of IBD after bacterial gastroenteritis, viral gastroenteritis, and parasitic gastrointestinal infections; the time period between the gastroenteritis episode and IBD diagnosis was also evaluated.

### “Gastroenteritis” Definition

For the purpose of this review, we have accepted gastroenteritis to include positive stool cultures (bacterial, viral, and parasitic pathogens), self-reported “gastroenteritis,” and from medical coding.

### Data Extraction and Quality Assessment

All articles retrieved from this search strategy were transcribed into a standardized form.

Relevant articles were initially selected on the basis of the title and abstract, after which the full text was read to confirm relevance. Data were extracted independently by 1 reviewer (A.D.), and checked independently by the second reviewer (Y.L.). Discrepancies were resolved by consensus between the 2 reviewers. If multiple publications of the same study were identified, then the most recent publication of data was included.

The quality of each study was then assessed by the 2 reviewers (A.D. and Y.L.) independently, using the modified Newcastle Ottawa Scale (NOS)^19^ for assessing the quality of nonrandomized studies in meta-analyses. For case–control studies, selection, comparability, and exposure were assessed. For cohort studies, selection, comparability, and outcome were assessed.

### Data Synthesis and Statistical Analysis

The summary statistics were derived from Review Manager version 5.3.^20^ For dichotomous data, Mantel–Haenszel method was used with a fixed effects model (if *I*^2^ less than 20), or a random effects model (if *I*^2^ greater than 20). Outcomes were recorded as odds ratios (ORs) with a 95% CI. *P* < .05 was the value set as statistically significant. Heterogeneity of studies was measured using *I*^2^.

### Ethical Considerations

The authors have no personal or financial conflicts of interest to disclose.

## Results

**Figure 1.**
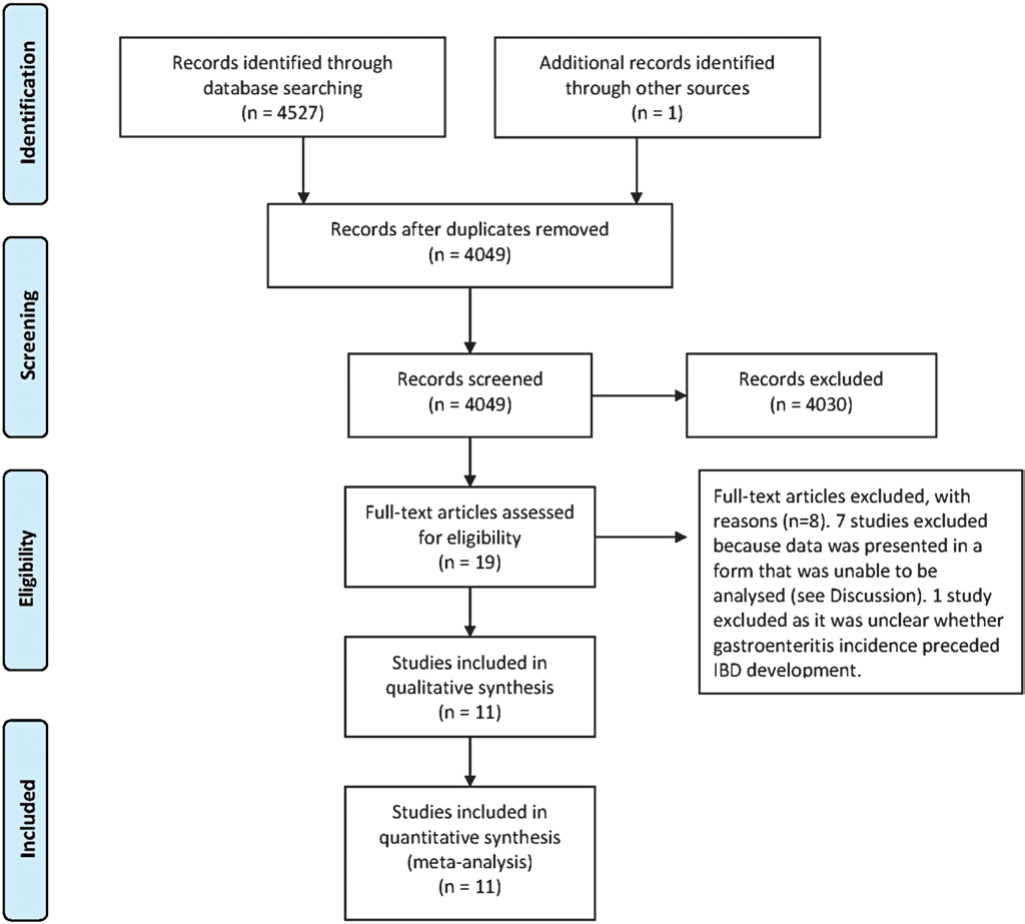
PRISMA diagram demonstrating search strategy for studies.

An initial search of the 2 databases revealed 1497 relevant articles. After review of abstracts and full texts, 11 studies (*n* = 923 608 patients) were identified that met our eligibility criteria.^21–31^ The search and selection process for the studies is depicted in a PRISMA flow diagram (Figure 1).

### Study Characteristics

The study characteristics are summarized in Table 1. Of the 11 studies included, 10 were case–control studies (with 2 being twin studies) and 1 was a cohort study. All studies included both CD and UC patients, with 2 studies also including IBD-unclassified patients. A variety of gastroenteritis subtypes were assessed; bacterial and unspecified gastrointestinal infections were the 2 most common (6 studies each).

**Table 1.** Study characteristics

Study, year	Country	No. participants	Study type	Gastroenteritis type	IBD subtype
Halfvarson, 2006^21^	Sweden/Denmark	634	Case–control (twin study)	Unspecified	CD/UC
Helms, 2006^22^	Denmark	52 121	Case–control	Bacterial	CD/UC
García Rodríguez, 2006^23^	United Kingdom	93 013	Case–control	Bacterial/unspecified	CD/UC/unclassified
Porter, 2008^24^	United States	14 665	Case–control	Bacterial/viral/protozoa/unspecified	CD/UC
Gradel, 2009^25^	Denmark	39 972	Case–control	Bacteria	CD/UC
López-Serrano, 2010^26^	Spain	783	Case–control	Unspecified	CD/UC
Jess, 2011^27^	Denmark	239 272	Cohort	Bacteria	CD/UC
Castiglione, 2012^28^	Italy	1557	Case–control	Parasitic	CD/UC
Ng, 2012^29^	United Kingdom	500	Case–control (twin study)	Unspecified	CD/UC
Chu, 2013^30^	South Africa	370	Case–control	Parasitic	CD/UC
Axelrad, 2019^31^	Sweden	480 721	Case–control	Bacterial/viral/parasitic/unspecified	CD/UC/unclassified

Abbreviations: CD, Crohn’s disease; IBD, inflammatory bowel disease; UC, ulcerative colitis.

### Risk of Bias

The NOS was used to assess the quality of the studies and is summarized in Table 2. There was variability in the quality of studies (score range 5–9). However, 5 of the studies did have low risk of bias, scoring 9/9 on the NOS.

**Table 2.** Risk of bias

Study, year	Selection (out of 4 stars)	Comparability (out of 2 stars)	Outcome/exposure (out of 3 stars)	Total
Halfvarson, 2006^21^	****	**	**	8
Helms, 2006^22^	****	**	***	9
García Rodríguez, 2006^23^	****	**	***	9
Porter, 2008^24^	****	**	***	9
Gradel, 2009^25^	****	**	***	9
López-Serrano, 2010^26^	****	**	**	8
Jess, 2011^27^	***	**	**	7
Castiglione, 2012^28^	***	—	**	5
Ng, 2012^29^	****	**	**	8
Chu, 2013^30^	***	—	**	5
Axelrad, 2019^31^	****	**	***	9

### Study Results

#### All gastroenteritis and IBD

**Figure 2.**
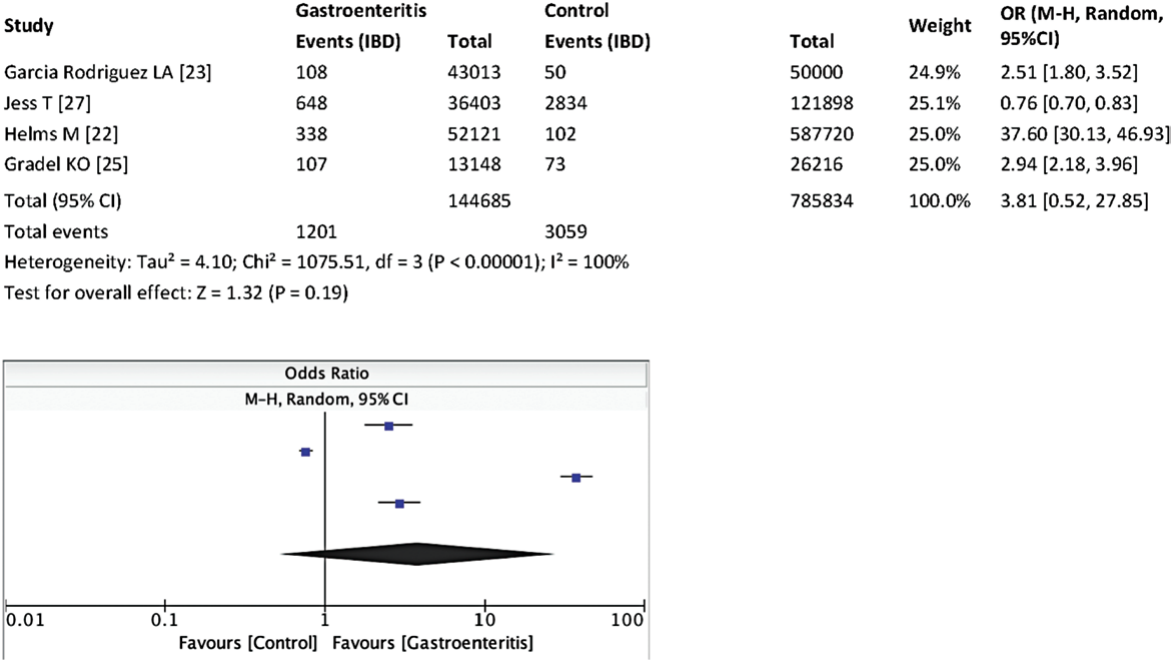
Incidence of IBD after gastroenteritis. Abbreviations: IBD, inflammatory bowel disease; OR, odds ratio.

Four studies assessed patients with gastroenteritis, subsequently developing IBD as the primary outcome.^22,23,25,27^ Patients with gastroenteritis had a higher incidence of IBD but this did not reach statistical significance (OR 3.81, 95% CI 0.52–27.85, *P* = .19, see Figure 2). There was also considerable heterogeneity across studies (*I*^2^ = 100%, see Discussion).

**Figure 3.**
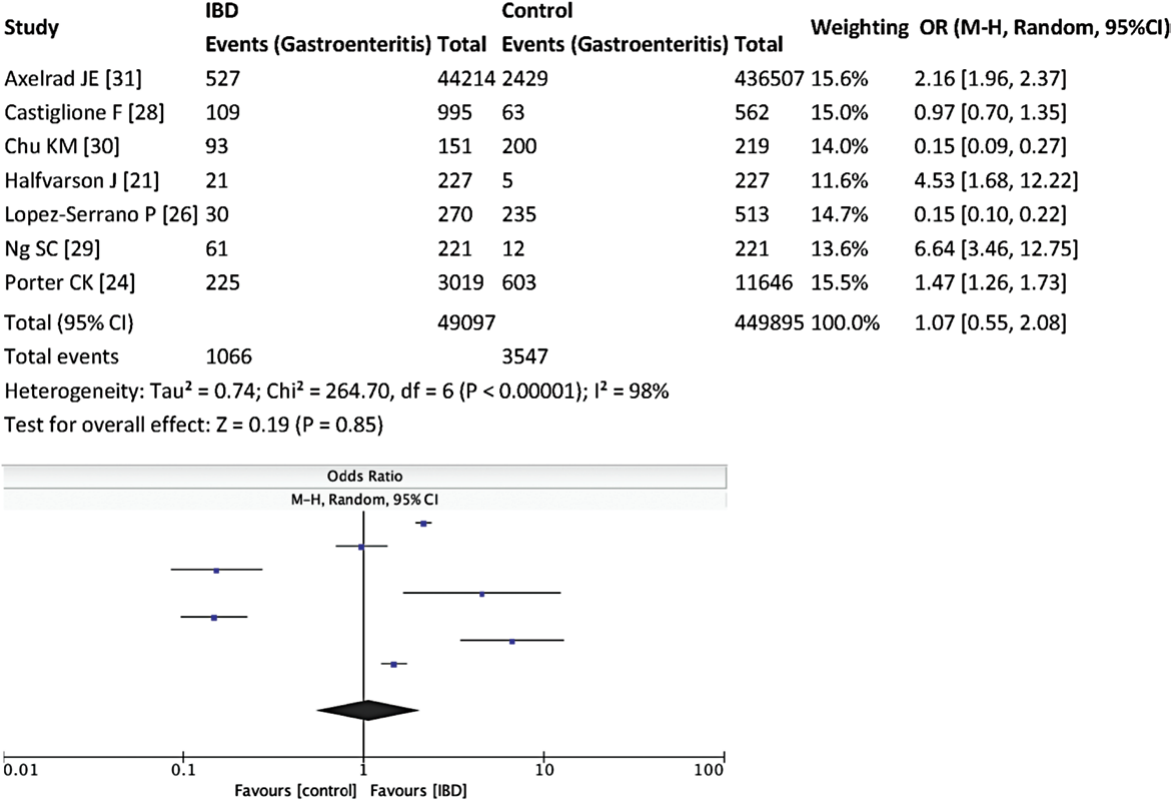
History of gastroenteritis in IBD patients versus controls. Abbreviations: IBD, inflammatory bowel disease; OR, odds ratio.

In addition, 7 studies examined the incidence of antecedent gastroenteritis (primary outcome) in patients with a confirmed diagnosis of IBD, compared to controlled population.^21,24,26,28–31^ There was no difference between incidence of antecedent gastroenteritis across the 2 population groups (OR 1.07, 95% CI 0.55–2.08, *P* = .85) (Figure 3), again with considerable heterogeneity (*I*^2^ = 98%). When excluding patients with parasitic infections,^28,30,31^ there was a trend toward increased likelihood of a previous gastroenteritis episode in patients with confirmed IBD, but this did not reach statistical significance (OR 3.60, 95% CI 0.20–65.60, *P* = .39).

Two of the studies were case-controlled “twin studies.” ^21,29^ When limiting analysis to these 2 studies alone, there was a significantly increased history of antecedent gastroenteritis in patients with IBD (OR 5.91, 95% CI 3.43–10.20, *P* < .00001); both studies performed logistic regression analysis to control for confounders.

#### Bacterial gastroenteritis and IBD

Six studies included patients with bacterial gastroenteritis, from which we were able to include 3 studies in a subanalysis.^22,25,27^ Patients with a history of bacterial gastroenteritis had a higher incidence of IBD, although this did not reach statistical significance (OR 4.38, 95% CI 0.34, 56.81, *P* = .26). One study^31^ included data on IBD patients who had a history of bacterial gastroenteritis compared to a control group, and found that IBD patients were more likely to have experienced this (adjusted OR 2.02, 95% CI 1.82, 2.24).

#### Viral gastroenteritis and IBD

Two studies did include patients with viral gastroenteritis,^24,31^ however only one of these detailed specifically the viral cases.^31^ In this study, viral gastroenteritis predicted a higher risk of IBD compared to control patients (adjusted OR 1.55, 95% CI 1.34–1.79).

#### Parasitic gastrointestinal infections and IBD

Only 3 studies detailed the risk of parasitic gastrointestinal infections and IBD development.^28,30,31^ There was considerable heterogeneity across the studies (*I*^2^ = 96%). One study showed no association between IBD and parasitic infection,^28^ 1 showed an increased associated between IBD and parasitic infection,^31^ and the third study showed a decreased risk of IBD associated with prior parasitic infection.^30^ Overall from our review, there was a trend toward a decreased risk of IBD associated with parasitic infection, but this did not reach statistical significance (OR 0.66, 95% CI 0.20, 2.15, *P* = .49).

#### Gastroenteritis and IBD subtype

There was no difference between patients with or without gastroenteritis and subsequent UC diagnosis (OR 1.76, 95% CI 0.67, 4.61, *P* = .25). Similarly, there was no difference between UC patients who reported a previous history of gastroenteritis and those who did not (OR 0.91, 95% CI 0.50, 1.67, *P* = .77). Similarly, there was no association between patients with gastroenteritis and the future development of CD (OR 1.82, 95% CI 0.54, 6.16, *P* = .34); also patients with CD had no increased association with antecedent gastroenteritis compared to controls (OR 1.03, 95% CI 0.56, 1.90, *P* = .91).

#### Specific pathogens and IBD

Several studies also examined risk of IBD with certain bacterial pathogens.^22,23,25,27,31^ Commonly studied microorganisms included *Salmonella*, *Campylobacter*, *Yersinia*, *Escherichia coli*, and *Shigella*. However, the literature was mixed regarding this. Four of the studies found an increased risk of IBD with *Salmonella* and *Campylobacter* infection^22,23,25,31^; however, Jess et al^27^ found that there was no association. Both Helms et al^22^ and Axelrad et al^31^ found that there was increased incidence of IBD with *Shigella*, *E. coli*, and *Yersinia* infection, with the latter study also finding an association with *Clostridium difficile* infection.

#### Time period between gastrointestinal infection and IBD diagnosis

Seven studies commented and/or assessed the time period between gastrointestinal infection and a diagnosis of IBD.^22–25,27,31^ Helms et al^22^ only included patients with diagnosis of IBD within 1 year post episode of gastroenteritis; conversely, Porter et al^24^ excluded patients with an episode of gastroenteritis within a 6-month time period before the first IBD diagnosis (to ensure an initial medical encounter was not a first presentation of undiagnosed IBD).

García Rodríguez et al^23^ found that the majority of patients (41%) had an interval 2–12 months between their gastroenteritis episode and IBD diagnosis; 34% had an interval of >24 months, and only 4% had an interval of 1 month. Gradel et al^26^ similarly found a steep increase in IBD incidence during the first year after *Salmonella*/*Campylobacter* exposure (and even after 15 years, with 1.2% incidence of IBD for those exposed to gastroenteritis compared to 0.5% to those unexposed). Axelrad et al^31^ also found that the time period between gastroenteritis and IBD remained a significant predictor for more than 10 years following an infectious episode (aOR 1.26; 1.19–1.33). Likewise, Jess et al^27^ found a steep increase in IBD diagnosis after *Salmonella*/*Campylobacter* infection during the first year postexposure, but this continued to be significantly increased in the 1–10 years after (although not after 10 years). Although the same study also found a steep increase in IBD diagnosis within 1 year after a negative stool test.

## Discussion

Our study has found that there is considerable heterogeneity in the literature regarding the role of gastroenteritis as a risk factor for IBD. Patients who had gastroenteritis had a higher incidence of subsequent IBD, but this did not reach statistical significance. There was also no significant difference found between IBD patients and a controlled population, in terms of incidence of antecedent gastroenteritis. Additionally, in our subanalysis, there was no association between IBD and bacterial, viral, or parasitic infections. These findings may be explained by the heterogeneity across studies, including country of origin as well as study methodology. Studies varied in their definition of gastroenteritis—several studies included stool cultures,^22,27^ self-reporting,^21,26,28–30^ and medical coding systems.^23–25,30^ Also there was variability in the types of gastrointestinal infections included in studies, with several studies being “unspecified.” ^21,26,29^

There has been suggestion in the literature that certain microbial pathogens may be associated with IBD pathogenesis, including *Mycobacterial avium paratuberculosis*,^32–34^*Helicobacter*,^35^*C. difficile*,^36^*E. coli*,^37^*Bacteroides*,^36^ and *Campylobacter*.^38^ Several viruses have also been postulated to be associated with IBD, including measles,^39^ EBV, and CMV.^40^ There has also been interest in the “hygiene hypothesis” as a contributor to the increasing incidence of IBD, with helminths possibly being an important immunoregulatory contributor to the gastrointestinal tract.^41^ Areas with higher helminthic intestinal infestation such as sub-Saharan Africa, have been associated with lower prevalence of IBD.^42,43^ Indeed, there have been studies investigating the role of helminths as an alternative therapy for IBD.^43^ However, the 3 studies in our review that included parasitic infections had conflicting results regarding association with IBD.^28,30,31^ Chu et al^30^ was the only study that found a protective factor associated with helminthic infections, in a South African population. However this study, as well as Castiglione et al^28^ relied on patient questionnaires to assess their primary outcome.

There is a relationship between commensal microorganisms in the gut microbiome and the mucosal immune system (MIS).^44,45^ Commensal microbes maintain a regulatory effect over the MIS and support the integrity of the intestinal epithelial cell (IEC) layer, resulting in mucosal homeostasis. Dysbiosis, as seen in gastroenteritis, diminishes the controlling influence of the commensal microbes, resulting in activation of the MIS with an inflammatory reaction and weakening of the IEC layer to invading pathogens. A healthy MIS is able to end the inflammatory response and restore IEC integrity, once the infection is controlled. It may be that individuals with a genetically defective MIS are unable to turn off the acute inflammatory response and proceed to a chronic IBD.

Although this systematic review found that patients who had gastroenteritis had a higher incidence of subsequent IBD, this was not statistically significant, possibly due to the high level of heterogeneity. However, is interesting to note that in the 2 case-controlled “twin studies,” ^21,29^ of low heterogeneity compared to other studies, had significantly increased history of antecedent gastroenteritis in patients with IBD when examined alone. No specific pathogen was identified.

Limitations of this study include the limited number of quality studies in the literature (see Table 2—NOS), as well as the considerable heterogeneity and variability of methodology data as already discussed. The heterogeneity was similarly noted in another systematic review published recently.^46^ However, our review differs in that we have specifically focused on IBD and the relationship with clinical “gastroenteritis,” as opposed to silent microbial pathogens. Additionally, patients may have been misdiagnosed with “gastroenteritis” when they actually had new-onset IBD, leading to confounding results. The reliance on patient questionnaires in several studies and the recall bias associated with this is another limitation^21,26,28–30^; infectious diarrhea has variability in clinical symptomatology that may also confound study interpretation. Furthermore, only few studies identified specific infectious pathogens in association with subsequent IBD development (as opposed to a diagnosis of “gastroenteritis” alone), with limited data precluding further detailed analysis. There was also variability in the assessment of the time interval between gastrointestinal infection and IBD diagnosis (with some studies not assessing this at all). There needs to be further research assessing the time relationship between initial gastrointestinal infection episode and subsequent IBD diagnosis. Studies also varied in the way they measured “gastroenteritis”; “recurrent gastrointestinal infections” as well as “hospitalizations” ^21^ or simply “more gastroenteritis.” ^29^

Additionally, antibiotic use is a well-known confounding factor in the relationship between gastroenteritis and risk of IBD—however this was only considered in 6 studies.^21,23,26,28,29,31^ Three studies found no association between IBD and antibiotic use.^21,23,28^ Two studies^23,31^ found increased risk of IBD with gastroenteritis treated with antibiotics (which may be associated with increased severity of gastroenteritis episode); and the final study found decreased risk.^26^

This review could also be affected by potential publication bias against negative studies. Other large case–control studies, not showing any correlation between gastroenteritis and IBD, may not have been accepted or even submitted for publication. This could lead to an overestimation of the role of gastroenteritis in the onset of IBD. At present, ‘funnel plots’ are used to estimate the extent of publication bias. However, it has been suggested that these funnel plots are inappropriate when the studies are heterogeneous, estimating different effects, like the studies used in our meta-analysis.^47^

The issue of detection bias was raised in 1 study. Jess et al^27^ was the only study to include incidence of IBD in patients positive stool cultures compared to those with negative stool cultures. They found that their incidence rate ratios (IRRs) for IBD were significantly higher after *Salmonella* or *Campylobacter* positive stool tests in the first year (IRRs 5.4–9.8) and thereafter. Interestingly however, IRRs for IBD <1 year after a negative stool test were several-fold higher in the first year (IRRs 53.2–57.5). They concluded that the similarities in the temporal risk profile for IBD following positive or negative stools tests may result from detection bias.

## Conclusions

In summary, our systematic review has shown that there is considerable variability in the literature regarding the role of gastroenteritis in the development of IBD. Patients who had gastroenteritis had a higher incidence of subsequent IBD, but this did not reach statistical significance. There was also no significant difference found between IBD patients and a controlled population, in terms of incidence of antecedent gastroenteritis. Further higher quality studies need to be performed to ascertain the true association between IBD and an infectious etiology.

## Data Availability

No new data were created, as this is a systematic review. However, specific details regarding the analysis of this preexisting data are available from the authors upon request.
